# Electrophysiological evidence for flexible goal-directed cue processing during episodic retrieval

**DOI:** 10.1016/j.neuroimage.2016.02.025

**Published:** 2016-05-15

**Authors:** Jane E. Herron, Lisa H. Evans, Edward L. Wilding

**Affiliations:** Cardiff University Brain Research Imaging Centre (CUBRIC), School of Psychology, Cardiff University, Cardiff CF10 3AT, Wales, UK

**Keywords:** Event-related potentials, Episodic memory, Cue processing, Retrieval orientation, Content specificity, Task-switching, Preparatory processing

## Abstract

A widely held assumption is that memory retrieval is aided by cognitive control processes that are engaged flexibly in service of memory retrieval and memory decisions. While there is some empirical support for this view, a notable exception is the absence of evidence for the flexible use of retrieval control in functional neuroimaging experiments requiring frequent switches between tasks with different cognitive demands. This absence is troublesome in so far as frequent switches between tasks mimic some of the challenges that are typically placed on memory outside the laboratory. In this experiment we instructed participants to alternate frequently between three episodic memory tasks requiring item recognition or retrieval of one of two different kinds of contextual information encoded in a prior study phase (screen location or encoding task). Event-related potentials (ERPs) elicited by unstudied items in the two tasks requiring retrieval of study context were reliably different, demonstrating for the first time that ERPs index task-specific processing of retrieval cues when retrieval goals change frequently. The inclusion of the item recognition task was a novel and important addition in this study, because only the ERPs elicited by unstudied items in one of the two context conditions diverged from those in the item recognition condition. This outcome constrains functional interpretations of the differences that emerged between the two context conditions and emphasises the utility of this baseline in functional imaging studies of retrieval processing operations.

## Highlights

• Three different episodic memory tasks were intermixed.
• Event-related potential correlates of unstudied retrieval cues were analysed by task.
• Neural correlates of these items differed according to retrieval task.
• Novel ERP evidence for flexible task-dependent processing of retrieval cues.

## Graphical abstract

**Figure:**
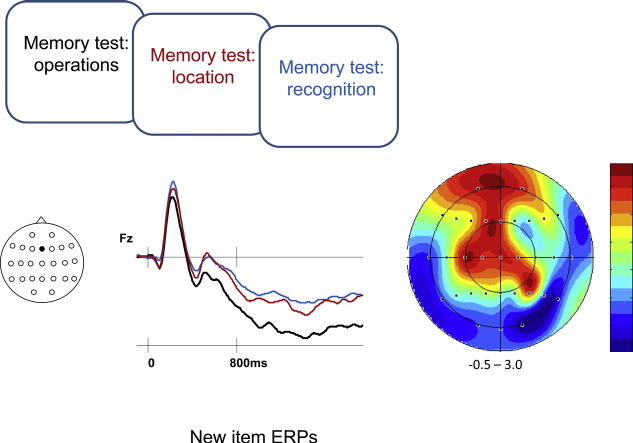

## Introduction

Episodic memory allows us to navigate our personal past and to recollect detailed information about specific events. Various models of episodic memory have assumed that this ability is enabled by control processes that specify and initiate memory searches, and process stimuli (either externally experienced or internally generated) in a way that maximises our ability to retrieve relevant information. Burgess and Shallice (1996) proposed a set of control processes involved in autobiographical recollection, one category of which (‘descriptors’) specify the memory search. Anderson and Bjork (1994) argued that recollection can be influenced by *cue bias* mechanisms which shape the nature of the memory search by influencing the way in which retrieval cues are processed. More specifically, they argued that recollection will suffer if the contextual representation specified as part of the memory search does not match the encoding context (*context bias*). In a similar vein, Mecklinger (2010) argued that cue-bias processes are applied to the internal representation of a retrieval cue in order to optimise the cue–memory trace interaction by constraining or specifying relevant cue features.

Rugg and Wilding (2000) introduced the term ‘retrieval orientation’ to encapsulate the concept that participants can adopt and maintain episodic retrieval sets that influence the processing of retrieval cues in ways that depend upon the specific retrieval requirements. They argued that contrasting neural activity elicited by unstudied items across memory tests that differ in their retrieval requirements will reveal differences in cue processing that are the consequences of having adopted content-specific orientations (for earlier related work, see Johnson et al., 1993, Wilding, 1999). One of the strengths of this contrast is that differences due to retrieval orientations are not confounded with differences between retrieved content, and this account has influenced a large number of studies designed to understand retrieval cue processing and its neural basis in a series of ERP (Robb and Rugg, 2002, Herron and Rugg, 2003, Hornberger et al., 2004, Hornberger et al., 2006a, Ranganath and Paller, 1999, Ranganath and Paller, 2000, Dzulkifli et al., 2004, Dzulkifli and Wilding, 2005, Bridger et al., 2009, Rosburg et al., 2011, Rosburg et al., 2013, Rosburg et al., 2014, Roberts et al., 2014) and fMRI studies (Hornberger et al., 2006b, Woodruff et al., 2006, Morcom and Rugg, 2012). Furthermore, there is evidence that these task-dependent differences in cue processing are associated both with increases in retrieval accuracy (Bridger et al., 2009, Bridger and Mecklinger, 2012, Roberts et al., 2014) and with the strategic recollection of task relevant information at the expense of less relevant information (e.g. Herron and Rugg, 2003, Dzulkifli and Wilding, 2005, Morcom and Rugg, 2012). It is therefore reasonable to assume that these effects index processes that influence memory retrieval directly.

One important finding that has been replicated in a number of ERP studies is that task-dependent differences in cue processing have only been observed when retrieval demands are blocked (i.e. when the entirety of each memory test retains the same retrieval demands), and that they are eliminated when participants are asked to make frequent switches between different memory tasks (Wilding and Nobre, 2001, Herron and Wilding, 2006, Johnson and Rugg, 2006, Werkle-Bergner et al., 2005). Wilding and Nobre (2001) asked participants to make Remember/Know judgments on the basis of whether they could remember either phonological or imagery-based associates from encoding, and found neural differences between correct rejections (correctly identified unstudied items) in the two tasks when they were blocked and not when they were mixed. Herron and Wilding (2006) cued participants trial-by-trial to make source memory decisions regarding either study location or encoding task, and found differences between correct rejections only when the tasks were predominantly blocked and not when they alternated frequently. Werkle-Bergner et al. (2005) reported task-dependent ERP differences between correct rejections in a general recognition task and a specific task regarding stimulus font when these tasks were blocked but not when they were mixed. Finally, Johnson and Rugg (2006) cued participants before each test item to identify whether the item had been studied either as a word or as a picture (different elaborative encoding tasks were also completed according to stimulus material), and found differences between correct rejections when these requirements were blocked as opposed to mixed. It has been stated on the basis of these consistent findings that retrieval orientations ‘develop over multiple trials and cannot be adjusted merely in response to an instructional cue’ (Johnson & Rugg, 2006, pp. 1531) and that ‘participants are unable to adjust their retrieval orientation on a trial by trial basis’ (Roberts, Tsivilis & Mayes, 2014, pp. 124).

The possibility that the engagement of certain classes of retrieval control process takes a number of trials to develop might be regarded as counter-intuitive, given that memory retrieval is something that is commonly accomplished among and in parallel with other cognitive tasks. Requirements to switch frequently between tasks, therefore, bear at least some similarities with the circumstances under which memory is often used. Moreover, the absence of ERP evidence of this kind is at odds with evidence from other sources that memory control processes are highly flexible. ERPs elicited by preparatory cues that direct participants to prepare to retrieve specific information about upcoming test items vary markedly despite frequent switches between cue-types (Herron and Wilding, 2004, Herron and Wilding, 2006). Moreover, Ecker and Zimmer (2009) reported that ERP correlates of familiarity were modulated by general versus specific retrieval orientations in a task-switching paradigm, and Koutstaal (2006) reported behavioural evidence that participants could flexibly switch between gist-based and specific retrieval orientations when cued trial-by-trial. These findings are consistent with the view that retrieval cues are subject to task-specific processing to some degree in task-switching paradigms. It is possible that ERP studies have thus far failed to detect these differences because they tend to be smaller in magnitude in mixed than in blocked paradigms.

This study was designed to maximise sensitivity to ERP differences in task-dependent retrieval cue processing within a task-switching paradigm. In order to enhance the likelihood of detecting differences elicited by ERPs associated by unstudied test items, retrieval of very different kinds of information was emphasised in two retrieval tasks. One task required the retrieval of elaborative encoding operations whereas the other required the retrieval of perceptual location-based information. This was the same task pairing used by Herron and Wilding (2006), but the paradigm was modified to further constrain participants' retrieval orientations. Preparatory cues started each test trial and varied frequently. The preparatory cues took the form of specific questions regarding encoding context which required simple yes/no answers. This was the approach taken by Johnson and Rugg (2006), but we predicted that combining this form of targeted cue with a pair of retrieval tasks that were more polarised in their contents would increase the likelihood of detecting evidence for flexible task-dependent cue processing.

A further development is the inclusion of a third task requiring item recognition only. A pairwise contrast between ERPs elicited by unstudied items in two specific retrieval tasks does not allow differences observed between the two to be ascribed to a particular task, or to determine whether the differences reflect the engagement of qualitatively different processes (indicative of content-specific processing) or quantitative differences between the same operations that are engaged across the two tasks (see Bridger et al., 2009, Bridger and Mecklinger, 2012, Roberts et al., 2014). Employing a general recognition baseline offers the potential for additional insights into the locus and the functional nature of differences detected between the two specific tasks, the assumption being that there is not an incentive to focus on specific contextual details to the same extent in the recognition task as in the other tasks.

Finally, the paradigm will also allow us to examine ERPs that index processes linked to the adoption of retrieval orientations. This will be achieved by time-locking ERPs to the onset of the preparatory cues indicating which retrieval task to complete (Herron and Wilding, 2004, Herron and Wilding, 2006). In direct contrast with the circumstances under which ERPs elicited by correct rejections have tended to differ, divergences between the ERPs elicited by these cues have been observed when retrieval tasks vary frequently, and not when retrieval tasks are blocked (Herron and Wilding, 2006). These outcomes suggest that the ERPs elicited by different preparatory cues should diverge in this experiment, and if this is accompanied by divergences between the ERPs elicited by new test items, it would offer – for the first time – an opportunity to consider the correspondence between neural signatures of two classes of process linked to retrieval orientations: those engaged during their adoption, and those that are a consequence of an orientation having been adopted.

## Material and methods

### Participants

Data from 16 participants (14 female) were included, and data from a further 3 participants were excluded because they failed to contribute at least 16 artefact free trials to the conditions of interest. All participants were right-handed native English speakers aged 18–22 (average 20 years). They were paid at a rate of £7.50/h and gave informed consent before participating.

### Design

Stimuli were 288 visually presented words (frequency range of 1–10/million, MRC psycholinguistic database, Coltheart, 1981). Each experiment list comprised twelve study-test cycles. Twelve items were presented at study in each cycle, and these were repeated during the subsequent test phase together with a further twelve unstudied items. No items were repeated across cycles. During each study phase, words were blocked into groups of 6. Words in one block required animate/inanimate judgments, while words in the other block required indoor/outdoor judgments. The presentation order of these encoding tasks was counterbalanced. In addition, half of the study words in each block were presented to the left of fixation and half to the right. During each test phase, test items were preceded by preparatory cues which directed participants to prepare to make yes/no memory decisions about the upcoming test item. Two of these cues required participants to retrieve information regarding encoding operations (‘Animacy?’ and ‘In/Out?’), two required them to retrieve information regarding encoding location (‘Left?’ and ‘Right?’) and a fifth cue required a recognition judgement (‘Old?’). Operations cues (‘Animacy?’ and ‘In/Out?’) preceded one third of test items, Location cues (‘Left?’ and ‘Right?’) preceded a further third of test items, and Recognition cues (‘Old?’) preceded the remaining third of test items. The order of these three retrieval tasks was pseudo-randomised, with the constraint that cue types pertaining to each retrieval task were presented for two consecutive trials after which cue types pertaining to a different retrieval task were shown for two consecutive trials. This structure rendered the first trial of each pair unpredictable to participants. The encoding tasks, the left/right location of study words and the old/new designation of words were all counterbalanced fully.

### Procedure

Stimuli were presented in white font on a black background, on a monitor 1.2 m from the participant. The stimuli subtended maximum visual angles of 0.5° (vertical) and 2.2° (horizontal). Each study phase required participants to attend to the left/right location of each item in addition to performing the relevant encoding task. The first encoding task was specified by an on-screen instruction presented for 2000 ms at the start of the block (‘Animate or Inanimate?’ or ‘Indoors or Outdoors?’), and participants performed this task until a second on-screen instruction presented for 2000 ms directed them to switch to the alternate encoding task half-way through each study phase. Study words were presented for 300 ms after which the monitor was blanked for 1500 ms. A fixation asterisk was then presented for 500 ms after which the screen was blanked for a further 200 ms before presentation of the next study word. Responses were made by key presses. In the animacy task, participants responded with one hand to words denoting animate entities and with the other hand to words denoting inanimate entities. In the indoors/outdoors task, participants responded with one hand to items generally found indoors, and with the other hand to items generally found outdoors. The mapping of hands to response types was counterbalanced across participants.

During the test phase, all stimuli were presented at fixation. Preparatory cues (300 ms duration) were replaced by an asterisk (2000 ms) and then the retrieval cue (300 ms) which comprised either a studied or an unstudied test word. The monitor was then blanked until a response was made, and remained blank for a further 500 ms before a fixation asterisk was presented for 1000 ms. The next preparatory cue was presented after the screen was blanked for a further 100 ms. Participants were instructed to attend to each preparatory cue in order to identify the retrieval question, and to respond to the subsequent retrieval cue accordingly. A yes/no response was required in all cases; participants responded with one hand if the test item was associated with the encoding context specified by the preparatory cue and with the other hand if it was not. The hands designated for these responses were counterbalanced across participants. Participants were asked to balance speed and accuracy equally, and to fixate centrally throughout.

### EEG acquisition

EEG was recorded from 32 recording locations based on the International 10–20 system (Jasper, 1958) including midline (Fz, Cz, Pz, Oz) and left/right hemisphere locations (FP1/FP2, F7/F8, F5/F6, F3/F4, F1/F2, T7/T8, C5/C6, C3/C4, C1/C2, P7/P8, P5/P6, P3/P4, P1/P2, O1/O2). Additional electrodes were placed on the mastoid processes. The electrooculogram (EOG) was recorded from above and below the left eye (vertical (V)EOG) and from the outer canthi (horizontal (H)EOG). The electroencephalogram (EEG; range DC-419 Hz; sampling rate 2048 Hz) was acquired referenced to linked electrodes located midway between POz and PO3/PO4, respectively, and was re-referenced off-line to the average of the signal at the mastoids. Event-related potentials were time-locked to the presentation of both preparatory cues and new (unstudied) test items. Trials containing large EOG artefact were rejected, as were trials containing A/D saturation or baseline drift exceeding 80 μV. Other EOG blink artefacts were corrected using a linear regression estimate (Semlitsch et al., 1986). A 7-point binomially weighted smoothing filter was applied prior to analysis. Data were filtered off-line (0.03–40 Hz) and down-sampled to 125 Hz, resulting in a total epoch length of 2048 ms with a 104 ms baseline relative to which all mean amplitudes were computed.

## Results

A weighted average of data associated with ‘Animacy?’ and ‘In/Out?’ cues (i.e. cues requiring the retrieval of encoding operations) was created and these task requirements will be referred to as *Operations*. A weighted average of data associated with ‘Left?’ and ‘Right?’ cues (those requiring the retrieval of encoding location) was created and these task requirements will be referred to as *Location*. Task requirements associated with the ‘Old?’ cue will be referred to as *Recognition*. Studied items eliciting correct ‘yes’ responses will be referred to as *Target Hits*, studied items eliciting correct ‘no’ responses will be referred to as *Nontarget Hits*, and unstudied items eliciting correct ‘no’ responses will be referred to as *Correct Rejections*. All analyses included the Greenhouse–Geisser correction for non-sphericity where necessary (Greenhouse and Geisser, 1959), and epsilon-corrected degrees of freedom are given in the text.

### Behaviour

Table 1 shows behaviour at test separated according to retrieval task.

ANOVA of correct responses incorporating the factors of Response Type (Target Hits/Nontarget Hits/Correct Rejections) and Retrieval Task (Operations/Location) gave rise to a main effect of Response Type (*F*_(*1.3*,*19.1*)_ = 48.12; *p* < 0.001) only. Subsidiary analyses revealed that accuracy associated with Correct Rejections was higher than that associated with both Target Hits (*F*_(*1*,*15*)_ = 33.95; *p* < 0.001) and Nontarget Hits (*F*_(*1*,*15*)_ = 101.13; *p* < 0.001) and that Target Hit accuracy was higher than Nontarget Hit accuracy (*F*_(*1*,*15*)_ = 22.05; *p* < 0.001). ANOVA of Target Hits and Correct Rejections from all three retrieval tasks incorporated the factors of Response Type (Hits/Correct Rejections) and Retrieval Task (Operations/Location/Recognition) and gave rise to a main effect of Response Type (*F*_(*1*,*15*)_ = 34.84; *p* < 0.001) that was moderated by an interaction with Retrieval Task (*F*_(*1.6*,*24.7*)_ = 4.30; *p* < 0.05). Subsidiary analyses showed that Correct Rejections did not differ across retrieval tasks, and that the only significant difference in Target Hit accuracy was between the Recognition and Location tasks (*F*_(*1*,__*15*)_ = 9.94; *p* < 0.01).

An analogous ANOVA of RTs associated with correctly classified items (Target Hits/Nontarget Hits/Correct Rejections) from the Operations and Location tasks gave rise to main effects of Retrieval Task (*F*_(*1*,*15*)_ = 10.59; *p* < 0.05) and Response Type (*F*_(*1.6*,__*23.6*)_ = 52.49; *p* < 0.001) as well as a Retrieval Task × Response Type interaction (*F*_(*1.7*,__*25.9*)_ = 5.54; *p* < 0.05). A significant effect of Retrieval Task was observed only for Nontarget Hits (*F*_(*1*,*15*)_ = 10.27; *p* < 0.01), with reaction times being slower in the Operations than in the Location task. Further subsidiary analyses confirmed that reaction times were generally longer for Nontarget than for Target Hits (*F*_(*1*,*15*)_ = 21.45; *p* < 0.001), which were in turn longer than Correct Rejections (*F*_(*1*,*15*)_ = 40.77; *p* < 0.001). The ANOVA of RTs associated with Target Hits and Correct Rejections from all three retrieval tasks gave rise to a main effect of Retrieval Task (*F*_(*1.9*,*28.3*)_ = 27.92; *p* < 0.001), a main effect of Response Type (*F*_(*1*,*15*)_ = 40.24; *p* < 0.001) and an interaction between these two factors (*F*_(*1.8*,*26.9*)_ = 21.54; *p* < 0.001). Reaction times to Target Hits were slower than to Correct Rejections, and reaction times to Correct Rejections did not differ according to retrieval task. Subsidiary analyses confirmed that reaction times associated with Target Hits were faster in the Recognition task than in both the Location task (*F*_(*1*,*15*)_ = 22.74; *p* < 0.001) and the Operations task (*F*_(*1*,*15*_) = 45.11; *p* < 0.001) while times for the latter two response types did not differ significantly.

### Event-related potentials

#### Correct rejections

**Fig. 1:**
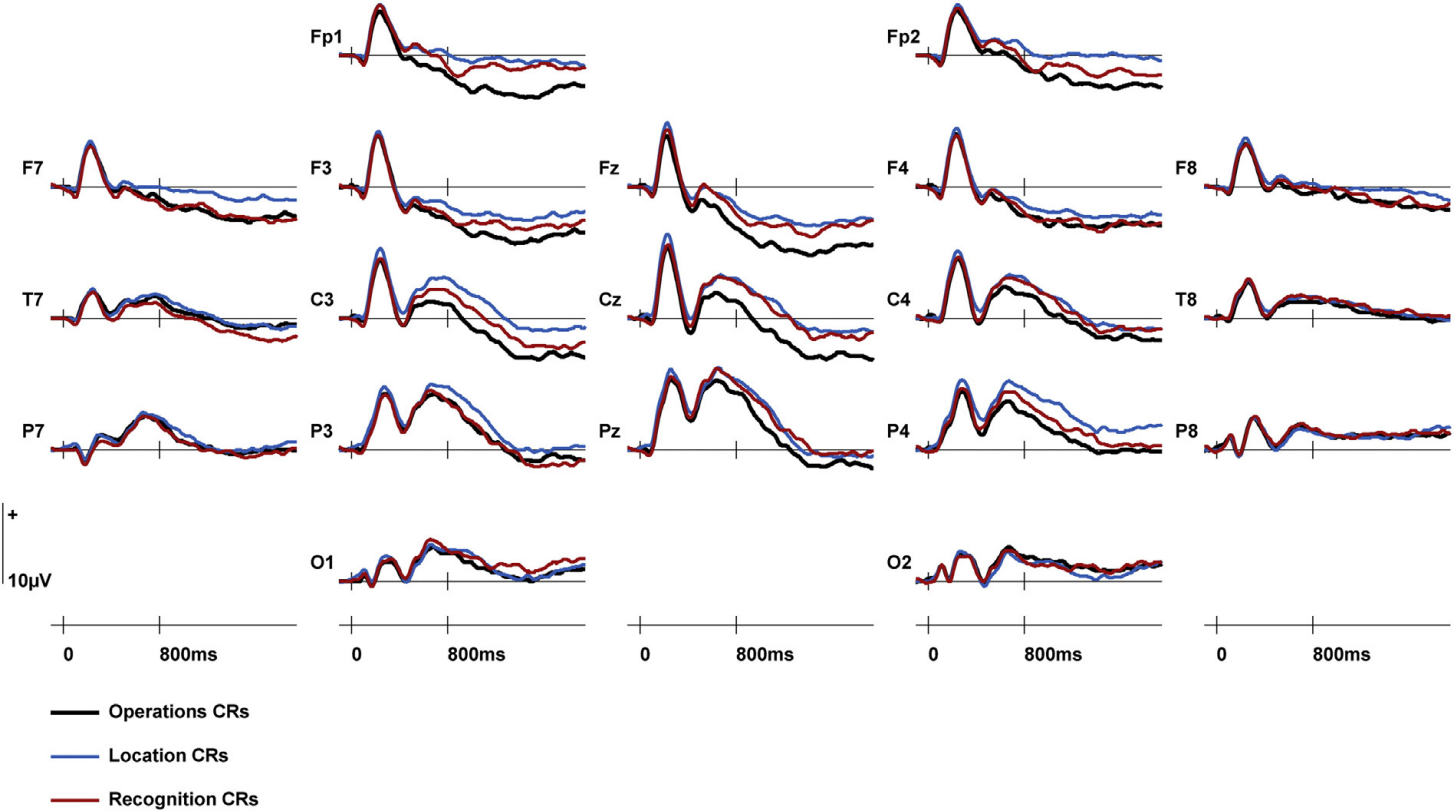
ERPs elicited by Correct Rejections (CRs) in each of the three retrieval tasks from frontopolar (Fp1, Fp2), anterior (F7, F3, Fz, F4, F8), central (T7, C3, Cz, C4, T8), posterior (P7, P3, Pz, P4, P8) and occipital (O1, O2) electrode sites.

Primary analyses were conducted upon ERPs elicited by Correct Rejections (CRs) separated according to retrieval task (Operations/Location/Recognition). Visual inspection of the data (see Fig. 1) indicated that task-related differences between these ERPs emerged at approximately 400 ms post-stimulus, taking the form of a slow wave which varied with retrieval task until the end of the recording epoch (1900 ms). These differences were widespread and largest towards the midline. Mean amplitudes of averaged ERPs were calculated for an a priori time window of 800–1900 ms guided by previous research showing effects of the same Operations/Location task pair on CRs in a blocked paradigm (Herron and Wilding, 2006). Mean ERP amplitudes from an earlier time window of 400–800 ms were also calculated due to the earlier onset of task effects in the present study. The mean numbers of trials (ranges in parentheses) contributing to each condition of interest were as follows: Operations CRs = 35 (19–48), Location CRs = 35 (20–49), Recognition CRs = 35 (16–46). ERPs within both the 400–800 ms and 800–1900 ms latency regions were measured at 24 sites distributed across the scalp (F1/F2, F3/F4, F5/F6, F7/F8, C1/C2, C3/C4, C5/C6, T7/T8, P1/P2, P3/P4, P5/P6, P7/P8). The initial global ANOVAs were conducted separately for each epoch and incorporated the factors of Retrieval Task (Operations/Location/Recognition), Anterior/Central/Posterior dimension, Hemisphere (left/right) and Site (inferior/mid-lateral/superior/midline).

**Fig. 2:**
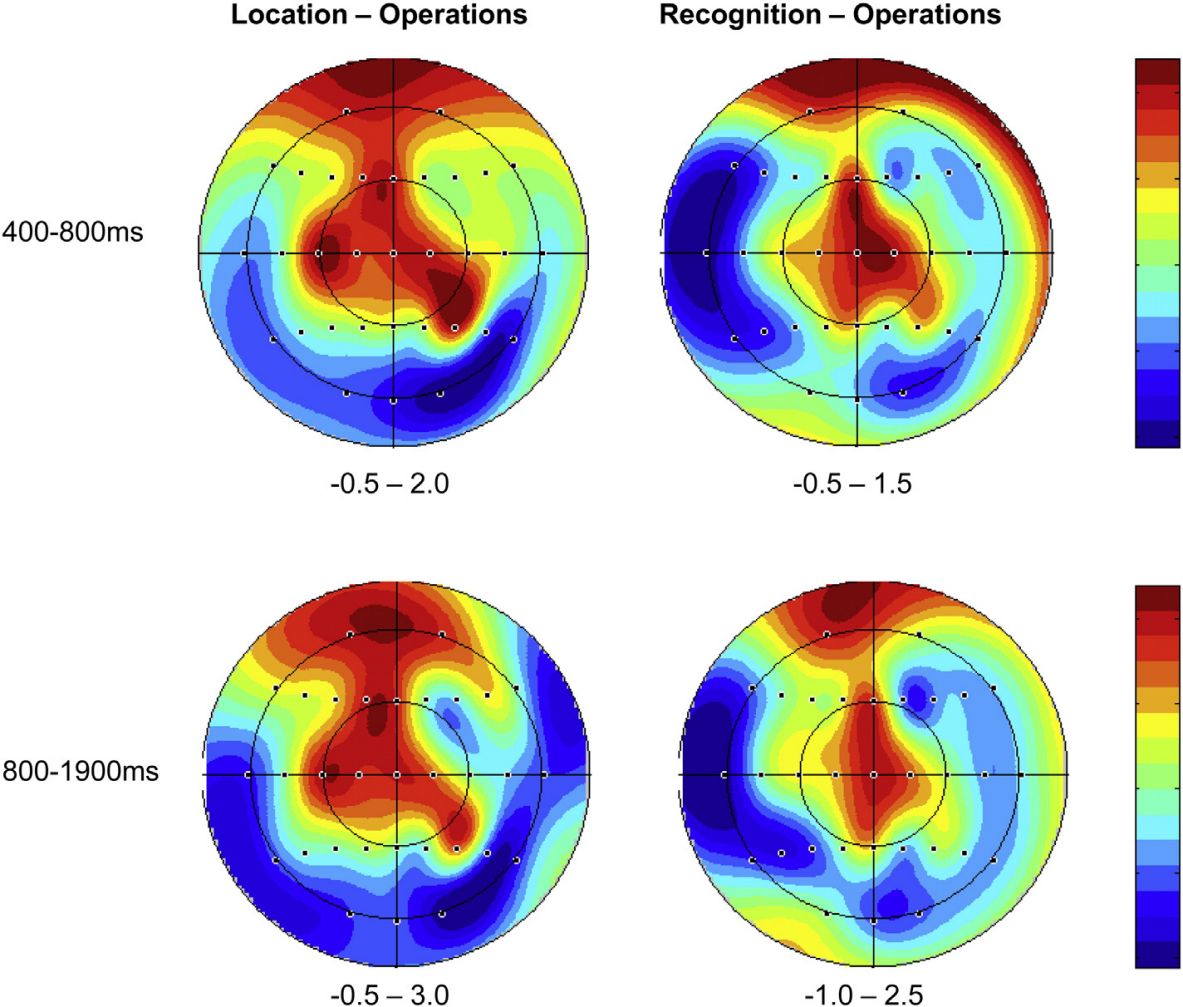
Scalp maps of retrieval orientation effects reaching statistical significance. Each map shows the scalp distribution of the effect obtained by subtracting Correct Rejection ERPs associated with the retrieval tasks specified above the map between 400–1900 ms. The scale bars to the right of each map show the amplitude of each effect in microvolts.

Analysis of ERPs from 400 to 800 ms revealed a Retrieval Task × Site interaction (*F*_(*2.4*,*35.3*)_ = 3.32; *p* < 0.05). Retrieval Task × Site interactions were also observed in pairwise comparisons between Operations CRs and Location CRs (*F*_(*1.4*,*20.6*)_ = 4.12; *p* < 0.05) and between Operations and Recognition CRs (*F*_(*1.3*,*19.9*)_ = 4.60; *p* < 0.05), due to greater negativity elicited by Operations CRs which was maximal towards the midline (see Fig. 2). No effects involving Retrieval Task were detected in the contrast between Location CRs and Recognition CRs.

Analysis of ERPs from 800 to 1900 ms also gave rise to a Retrieval Task × Site interaction (*F*_(*3.1*,*46.3*)_ = 3.11; *p* < 0.05). Pairwise comparisons revealed Retrieval Task × Site interactions in the contrasts between Operations CRs and Location CRs (*F*_(*1.9*,*28.2*)_ = 4.37; *p* < 0.05) and between Operations CRs and Recognition CRs (*F*_(*1.7*,*26.2*)_ = 4.75; *p* < 0.05). Both interactions reflected greater negativity for Operations CRs which became larger in amplitude towards the midline sites (see Fig. 2). No significant effect of Retrieval Task was detected in the contrast between Location CRs and Recognition CRs (F < 1).

Topographic analyses were conducted to determine whether the Operations/Location ERP effects differed qualitatively from the Operations/Recognition ERP effects, and whether these effects also differed qualitatively across the two time windows. This analysis was conducted on difference scores obtained by subtracting mean amplitudes of Operations CR ERPs from Location CR and Recognition CR ERPs respectively from the 24 sites included in the first stage analyses for the 400–800 ms and 800–1900 ms epochs. The data were rescaled using the max–min method to avoid confounding changes in amplitude with changes in the shape of scalp distributions (McCarthy and Wood, 1985), and the resulting ANOVA included the factors of Epoch (400–800 ms; 800–1900 ms), Condition (Location–Operations; Recognition–Operations), Anterior/Central/Posterior dimension, Hemisphere (left/right) and Site (inferior/mid-lateral/superior/midline). No effects involving Epoch and/or Condition were observed.

#### Preparatory ERPs

**Fig. 3:**
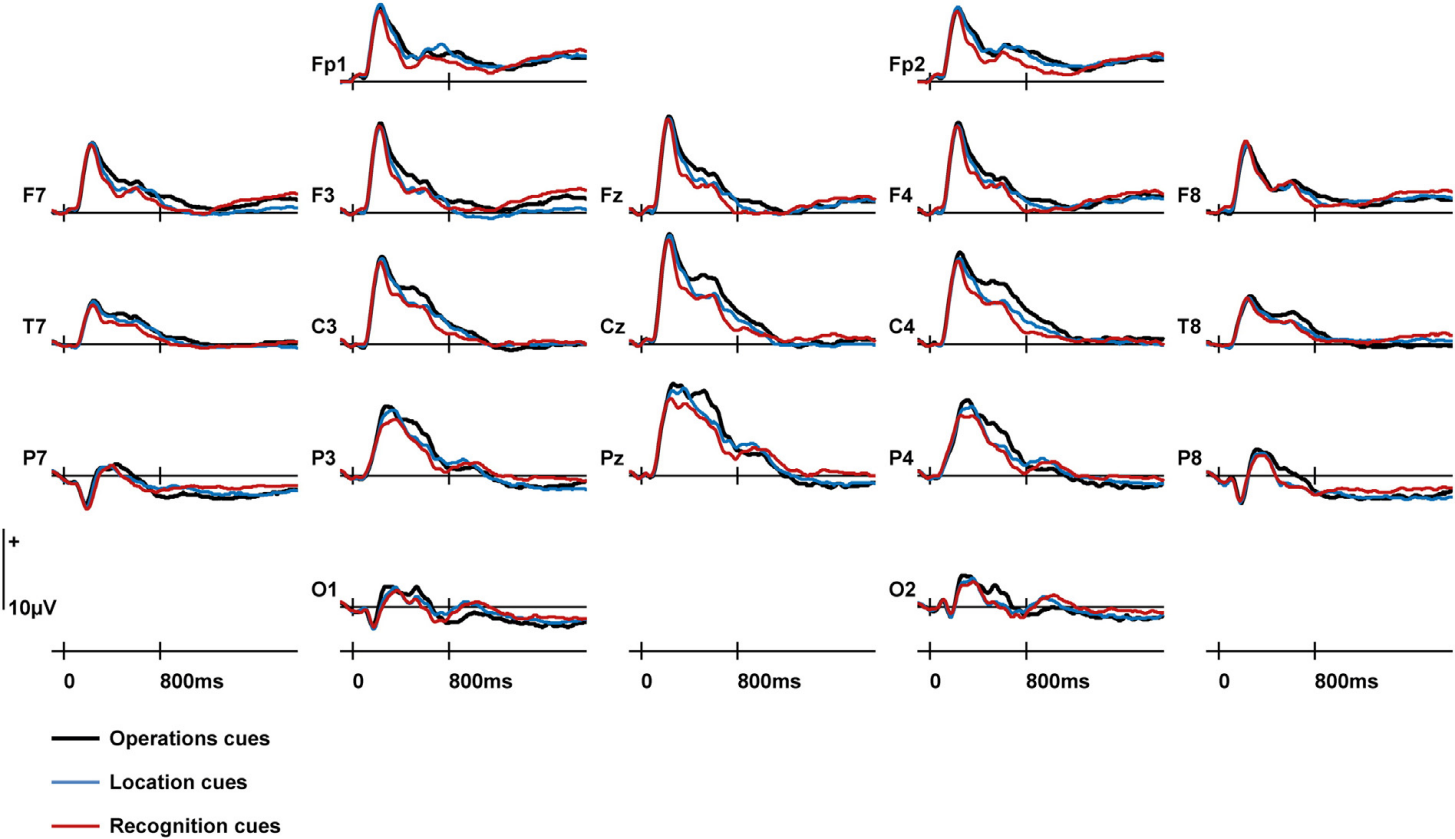
ERPs elicited by preparatory cues in each of the three retrieval tasks from frontopolar (Fp1, Fp2), anterior (F7, F3, Fz, F4, F8), central (T7, C3, Cz, C4, T8), posterior (P7, P3, Pz, P4, P8) and occipital (O1, O2) electrode sites. These data are a weighted average of ERPs elicited on switch and on stay trials.

A complementary set of analyses was conducted upon ERPs elicited by preparatory cues for the three tasks. Mean amplitudes were calculated for an a priori time window of 700–1900 ms, following Herron & Wilding (2006). Mean ERP amplitudes from an earlier time window of 200–700 ms were also calculated because preparatory ERPs appear to diverge during this period (see Fig. 3). In contrast with the data for new test items, there were sufficient trial numbers to separate the preparatory cue data according to switch/stay trial status. The mean numbers of trials (ranges in parentheses) contributing to each condition of interest were as follows: Operations Cues Switch = 37 (27–46), Operations Cues Stay = 37 (23–46), Location Cues Switch = 39 (26–47), Location Cues Stay = 38 (22–48), Recognition Cues Switch = 37 (26–47), and Recognition Cues Stay = 38 (25–48). The analyses for both epochs included the Switch/Stay factor, and this was the only difference between the factors included in this ANOVA and the one employed in the preceding section examining correct rejections. To anticipate the results, no effects of Switch/Stay were observed, and the preparatory ERPs shown in Fig. 3 are therefore a weighted average of ERPs on switch and on stay trials.

There were reliable effects including the factor of Retrieval Task for the 200–700 ms epoch only. These were a main effect of Retrieval Task (*F*_(*1.3*,__*20.1*)_ = 5.82, *p* < 0.05) and a Retrieval Task × Site interaction (*F*_(*2.5*,*36.9*)_ = 5.21, *p* < 0.05). Pairwise comparison of Operations and Location cues revealed a main effect of Retrieval Task (*F*_(*1*,*15*)_ = 4.65, *p* < 0.05) and an interaction between Retrieval Task, the Anterior/Posterior dimension and Hemisphere (*F*_(*1.9*,*28.5*)_ = 4.60, *p* < 0.05), reflecting relatively greater positivity for Operations cues that was maximal at right central and posterior sites. Pairwise comparison of Operations and Recognition cues revealed a main effect of Retrieval Task (*F*_(*1*,*15*)_ = 28.70, *p* < 0.001) and two interactions: Retrieval Task × Site (*F*_(*1.4*,*20.8*)_ = 10.88, *p* < 0.01) and Retrieval Task × Hemisphere × Site (*F*_(*2.0*,*30.3*)_ = 4.48, *p* < 0.05), reflecting a greater relative positivity for Operations cues that was maximal at right hemisphere sites closest to the midline. Mirroring the findings for the ERPs elicited by new test items, no effect of Retrieval Task was observed in the contrast between Location and Recognition cues.

## Discussion

For the first time significant differences were found between ERPs elicited by Correct Rejections across retrieval tasks between which frequent switches were required. These differences onset around 400 ms and were sustained until the end of the recording epoch (1900 ms). They took the form of a negative slow wave in the Operations task relative to the Location and Recognition tasks, and the magnitude of this effect increased towards the midline.

The behavioural data support the view that these divergences were driven by different retrieval demands rather than broader factors such as task difficulty. Overall response accuracy associated with Target Hits was high, and this measure was statistically equivalent both in the two context retrieval tasks (Operations and Location) and in the Recognition and the Operations tasks. Reaction times associated with Target Hits were also equivalent in the two context retrieval tasks. Both of these were longer than Target Hit reaction times in the Recognition task, which is unsurprising given the additional requirement to retrieve and evaluate source information in the context tasks. Reaction times associated with Correct Rejections were equivalent across all three retrieval tasks. There is therefore little reason to believe that participants found either of the two context retrieval tasks more difficult than the other.

A key question arising from these findings is why were participants able to flexibly adjust their retrieval orientations in the present study when no index of task-dependent retrieval cue processing was evident in Herron and Wilding's (2006) comparable mixed retrieval task? Although some ERP studies that have not found evidence of flexible retrieval orientations have employed longer study-test blocks and longer delays (e.g. Johnson and Rugg, 2006, Wilding and Nobre, 2001), this aspect of the experimental paradigm was highly similar between our previous study and the present one, and is therefore unlikely to account for this disparity (notably, Werkle-Bergner et al., 2005 also employed multiple short study-test blocks with short delays). The principal difference between the two experiments lies in the nature of the preparatory cues used to direct retrieval. Whereas the cues employed previously consisted of arbitrary symbols (X or O) requiring a three-way response (source 1/source2/new), the cues presented here took the form of questions regarding a specific encoding context requiring yes/no responses. The intention of using these cues was to help participants to constrain their retrieval orientations. The fact that Target Hit responses were significantly faster than Nontarget Hit responses confirms that participants did focus their retrieval efforts on retrieving the encoding context specified by the cue, only responding to Nontargets once they were confident that they were not associated with the target context. There is converging fMRI evidence using multivoxel pattern analysis that new items elicit increased activity associated with the target source when memory instructions encourage participants to focus on a single ‘target’ source rather than when instructions require participants to respond differentially to different sources (these instructions were blocked), and that memory accuracy for items from the target source is higher under targeted memory instructions (McDuff et al., 2009).

Similarly, there is some behavioural evidence that the constrained retrieval orientations demonstrated here benefited retrieval when compared with data from our previous study, with retrieval accuracy in both of the specific tasks being approximately 10% higher here than in Herron and Wilding (2006). Although this is consistent with the view that differences observed between ERPs in the two tasks reflect the engagement of task-dependent retrieval cue processes that facilitate the retrieval of task-relevant information, further work is required to replicate this link between memory accuracy and the presence/absence of ERP indices of retrieval orientation within a single study. Such a finding would reinforce those from other studies that have reported correlations between ERP indices of task-dependent retrieval cue processing and levels of retrieval accuracy (Bridger et al., 2009, Bridger and Mecklinger, 2012). As in the present study, Bridger et al. (2009) and Bridger and Mecklinger (2012) used retrieval paradigms that required participants to either endorse or reject items as belonging to a specific encoding context, which indicates that these kinds of paradigms may be more sensitive to variations in retrieval orientation than those with more general retrieval requirements.

Johnson and Rugg (2006) also used constrained cues of the kind employed here, yet did not detect any ERP differences in retrieval cue processing when frequent alternation between tasks was required. One explanation for this discrepancy is that their contrast was based largely on material differences at study (i.e. words versus pictures), although subjects could also make use of different elaborative encoding tasks performed according to the format of study items. Items presented as pictures were encoded in a size judgement task whereas items studied as words were encoded in an indoors/outdoors task. Although ERPs elicited by correct rejections have consistently differed according to picture/word format at encoding in blocked designs (Herron and Rugg, 2003, Hornberger et al., 2004, Hornberger et al., 2006a, Stenberg et al., 2006), it is possible that this effect is too small to be detected in a task-switching design. Alternatively, it may be the case that material-specific orientations are less easily adjusted on an item-by-item basis than the orientations elicited here.

The contents of the context tasks used here were designed to be more polarised than those employed previously, with tasks encouraging an elaborative encoding orientation contrasted with tasks encouraging a perceptual (location-based) orientation. Werkle-Bergner et al. (2005) also failed to find differences between correct rejections across two tasks which required general old/new recognition judgments and specific same/different font judgments when these tasks were mixed within test blocks. Given the perceptual demands of the specific task they employed, this finding is consistent with our failure to find robust differences in magnitude between the correct rejections in the recognition task and the specific task requiring the retrieval of study location. Our data show that the requirement to retrieve encoding task was responsible for the largest task-dependent differences in retrieval cue processing, as correct rejections in the Operations task diverged from those in the other two retrieval tasks. These retrieval requirements arguably provide a greater opportunity for recapitulating processes brought to bear upon the cue at study than the requirement to retrieve perceptual information intrinsic to the cue.

It is worth emphasising that this level of functional claim was enabled via the inclusion of the item recognition task alongside the Operations and Location tasks. This three-task approach has not, to our knowledge, been employed in this way before, and it adds functional leverage. There is, however, a pragmatic cost to this approach. The use of three retrieval tasks (recognition; operations; location) and the consequences for trial numbers per condition of interest meant that we were unable to analyse ERPs separated by specific cue-type (left; right; animacy; in/out) or according to whether items were presented on the first (switch) or second (stay) trial of each task. With respect to the switch/stay manipulation, the fact that we have reported divergences between ERPs elicited by correct rejections in a mixed retrieval task paradigm provides important new knowledge about the neural correlates of retrieval orientation and the flexibility of retrieval processing operations. Replicating the current study without the Recognition condition (and with an unpredictable sequence) would permit a separation of data on switch and on stay trials, and would have the present outcomes as a useful baseline. A similar approach could be taken to examine whether more finely grained differences between specific cue-types (e.g. animacy versus in/out) can be obtained in a mixed context.

The spatiotemporal characteristics of the Operations/Location effect observed here are highly similar to those reported by Herron and Wilding (2006) in their contrast of the same retrieval task pair in a blocked paradigm, indicating that this index of task-dependent cue processing was successfully replicated within an alternating task paradigm. Our findings therefore constitute compelling new evidence that participants can alternate between different retrieval orientations when tasks are mixed and adjust the processing of retrieval cues accordingly. The effect observed here onset approximately 400 ms earlier than the effect reported by Herron and Wilding (2006), and it is reasonable to suppose that the constrained preparatory cues enabled participants to engage task-relevant cue specification processes more quickly here than in our previous study. This onset time preceded averaged reaction times associated with retrieval success (Target Hits) in the Operations task by approximately 1500 ms, which supports the characterisation of this effect as a correlate of processes that contribute to memory judgments. Differences between cue specification processes across tasks have been broadly characterised as differences in retrieval orientation via the mechanism of *cue bias*. According to this account, a specific retrieval requirement requires the adoption of an appropriate retrieval orientation which influences the processing of subsequent retrieval cues so as to enhance the likelihood of retrieving task-relevant information, possibly by maximising the overlap between cue processing at study and at test. Topographic analyses indicated that the neural generators underlying this effect did not change throughout the recording epoch, which is consistent with previous findings that temporally extended orientation effects do not exhibit changes in distribution (e.g. Hornberger et al., 2004, Dzulkifli et al., 2004, Bridger et al., 2009). This has informed the functional role assigned to the effect, with the extended time course being consistent with proposals that ERP differences across tasks reflect the online maintenance of an internal representation of the cue that biases retrieval towards the relevant memory content (Hornberger et al., 2004, Bridger et al., 2009).

In taking this work forward, it may well prove useful to supplement careful control over trial sequences and task demands, as already described, with additional behavioural measures. In the memory for foils paradigm (Jacoby et al., 2005) it has been shown that the retrieval task demands under which new items are processed influence how memorable they will be on a subsequent retrieval task. It is reasonable to assume that the evidence of differential processing of new items in ERP studies will have consequences for the representation of those new items in memory and consequences for how they will be processed subsequently. A combination of direct ERP measures of the processing afforded new items, along with subsequent behavioural measures along the lines of the memory for foils paradigm, offers a potentially powerful means of exploring in detail the influences retrieval orientations have on representations, processing operations and behaviour.

Analyses were also conducted on the preparatory cues signalling the upcoming task to determine the correspondence between these data, signalling the initiation of a retrieval orientation, and the data associated with correct rejections demonstrating the consequences of adopting an orientation. In a previous study, Herron and Wilding (2006) observed reliable ERP differences when participants prepared to retrieve Operations versus Location information from 700 to 1900 ms post-cue. However, comparable outcomes were not obtained within the same time window in this experiment. Reliable differences were instead found in the preparatory data between 200 and 700 ms and these were invariant across the switch/stay manipulation. Moreover, divergences were detected between the same retrieval task pairs (Operations vs Location; Operations vs Recognition) as were found when examining the ERPs elicited by new test items. This is, to our knowledge, the first report of effects within the same experiment that may index processes linked to preparation to retrieve, as well as the consequences of doing so (although see Duzel et al., 1999 for related effects linked to retrieval success).

There are a number of potential reasons for the disparity in findings between this study and Herron and Wilding (2006). The first is the inclusion of the recognition baseline in this experiment, which altered the retrieval demands in terms of which tasks participants were required to switch among. The second is the more targeted nature of the cues and task demands in this experiment. This may have allowed participants to adopt and constrain orientations more rapidly than when a broader range of retrieval responses are required.

Some support for the view that these factors are potentially important in finding early preparatory cue effects comes from an earlier study by Herron and Wilding (2004). Here participants switched between preparing for and completing three tasks, two of which were the same as in this study: Location and Operations, and a third non-episodic task. An early preparatory effect (300–600 ms) was found that was invariant across the switch/stay dimension. There are a few commonalities between this study and the current one. The first was the requirement to switch between two source memory tasks and a third task; a recognition task here, and a semantic memory task in Herron and Wilding (2004). The second is that the preparatory cues associated with the different retrieval tasks were physically different both in the present study and in Herron and Wilding (2004). Due to the requirement to switch between three tasks these cues were more targeted and explicit (e.g. ‘Animacy?’) as opposed to the abstract cues that are usually used in these sorts of experiments which can be counterbalanced e.g. ‘X’ and ‘O’ (Herron and Wilding, 2006). These observations emphasise the importance of replicating the effects reported here in similar experiments with the factors highlighted above examined experimentally.

Despite these issues which require clarification, the combination of reliable divergences for preparatory cues and for new test items is encouraging. The fact that differences in neural activity associated with preparation for retrieval were observed between the same task pairs as differences in neural activity associated with the processing of test items suggests a correspondence that is worthy of further investigation, with a view to delineating the links between preparing for, and enacting, retrieval processing operations.

In conclusion, our main findings show that combining constrained preparatory cues with a highly distinct pair of retrieval tasks is sufficient to obtain ERP evidence of task-dependent retrieval cue processing in a task-switching paradigm. This novel demonstration that participants can flexibly adjust task-dependent retrieval cue processes is an important development in the study of retrieval control. The inclusion of a recognition baseline task provided new functional insights, revealing that the ERP orientation effect was largely driven by the requirement to retrieve encoding operations. The time course of the effect reported here is consistent with the view that it reflects cue processing operations related to memory search.

## Figures and Tables

**Table 1:** Response accuracy and associated RTs (ms) in each of the three memory tasks (standard deviations in brackets).

	Accuracy	RTs
*Operations*		
Target Hits	.88 (.14)	1965 (652)
Nontarget Hits	.68 (.14)	2496 (752)
Correct Rejections	.97 (.08)	1172 (325)

*Location*		
Target Hits	.86 (.09)	1716 (708)
Nontarget Hits	.70 (.14)	2007 (705)
Correct Rejections	.98 (.03)	1169 (279)

*Recognition*		
Hits	.94 (.05)	1189 (328)
Correct Rejections	.98 (.02)	1167 (304)
